# Wegener's granulomatosis presenting as an abdominal aortic aneurysm: a case report

**DOI:** 10.1186/1757-1626-2-9346

**Published:** 2009-12-18

**Authors:** Rajaraman Durai, Reshma Agrawal, Kim Piper, Karim Brohi

**Affiliations:** 1 Department of Vascular Surgery, The Royal London Hospital, Whitechapel Road, Whitechapel, London, E1 1BB, UK; 2 Department of Pathology, The Royal London Hospital, Whitechapel Road, Whitechapel, London, E1 1BB, UK

## Abstract

**Introduction:**

Aortic aneurysm is not common in young patient. When a young patient presents with abdominal aortic aneurysm, there may be an underlying cause.

**Case presentation:**

Here, we describe a case of a 33-year-old gentleman who presented with flu like illness, chest and abdominal pains following a tooth extraction. A chest X-ray and subsequent computerised tomogram of the chest and abdomen demonstrated lung nodules and an abdominal aortic aneurysm. The aneurysm was repaired and his serology was positive for Wegener's granulomatosis. A nasal mucosal biopsy confirmed WG. He was treated with oral steroids and cyclophosphamide. His graft leaked and had to be replaced with a synthetic graft. Two months after his re-operation, he remains well.

**Conclusion:**

Whenever a young patient presents with an abdominal aortic aneurysm, an underlying connective disease should be excluded because early steroid/immunosuppressive treatment may prevent the development of further aneurysms.

## Introduction

A young patient presenting with an abdominal aortic aneurysm is extremely rare. Here, we describe a case of a young male who presented with flu like illness, chest and abdominal pains following a tooth extraction. Investigations revealed an abdominal aortic aneurysm associated with Wegener's granulomatosis (WG). There are only two previous case reports in the literature on abdominal aortic aneurysm due to WG [1]. Hence our case is the third of its kind. This case is peculiar because only nasal biopsy confirmed the disease.

## Case presentation

**Figure 1:**
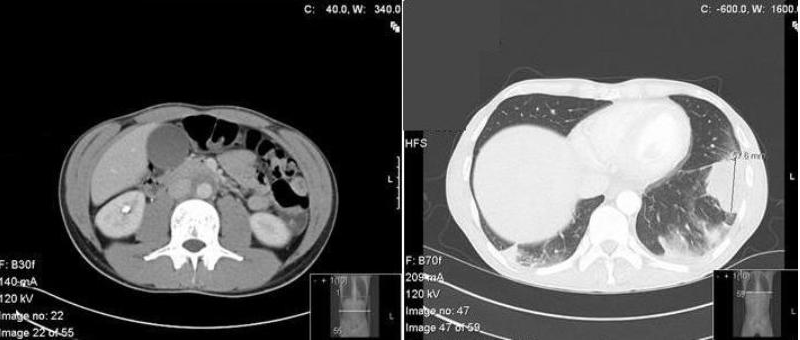
**(A) CT scan of abdomen showing a localised abdominal aortic aneurysm**. **(B) **CT scan of the chest showing lung nodules at the left lower lobe.

A 33-year-old Caucasian male presented with a history of being unwell, non-productive cough and constant abdominal discomfort in the upper abdomen for three weeks. All these symptoms started after a tooth extraction. There was no change in his bowel or bladder habits. Apart from clipping of sub arachnoid aneurysm 7 years previously, he did not have any significant past medical history. His pulse rate was 100 beats/minute and blood pressure was 110/70 mm Hg. The SaO2 and temperature were within the normal limits. Clinical examination showed bronchial breathing in the left base and some tenderness in the epigastric region without rebound or guarding. His blood tests showed an elevated white cell count of 14,000/μl and a C-reactive protein of 88 mg/dl. The rest of the blood test results were normal. A Chest X-ray showed opacity at the left lung base. Subsequently a computed tomogram (CT) of chest and abdomen was organised which demonstrated lung nodules at the left lung base with some cavitation and a small infra-renal abdominal aortic aneurysm (Figure 1).

Initially it was thought that the aneurysm was mycotic from his left lung abscess. The patient underwent bronchoscopy which was not successful. The aneurysm was excised partially and repaired with an internal jugular vein (IJV) graft. The aneurysm wall was sent for histology but due to a portering error the sample never reached the laboratory. The patient was discharged home on the 6^th ^post-operative day but he returned with severe abdominal pain on the seventh post operative day. A CT scan showed free fluid in the abdomen. The patient underwent re-laparotomy which revealed a hole in the IJV graft. The IJV graft was removed and replaced with an aorto-iliac silver impregnated synthetic trouser graft. During this time bloods were sent off for connective tissue screening which was positive for anti-proteinase PR3 (>1/10). Histology of a CT guided lung biopsy showed only necrotic tissue. His CRP and WCC remained high, but his blood cultures and aortic tissue never grew any bacteria. Therefore a nasal mucosal biopsy was organised which confirmed the presence of Wegener's granulomatosis.

**Figure 2:**
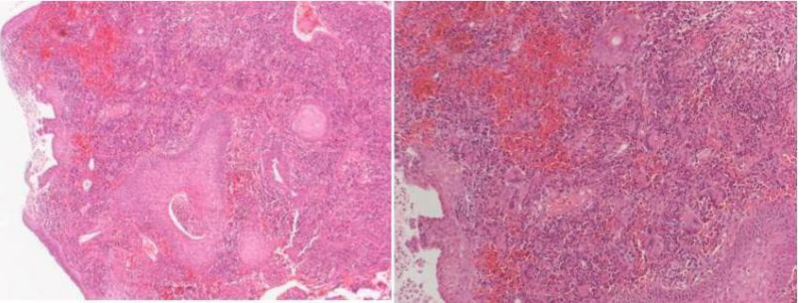
**(A) Haematoxylin and eosin (H&E) staining (× 10) of nasal biopsy showing mucosal ulceration (B) H&E (× 40) showing extensive inflammatory reaction in the corium, with hyperplastic rete processes, and giant cells**.

Microscopic examination of the nasal mucosa showed fibro vascular tissue which was partially covered by stratified squamous epithelium and extensively ulceration. There was acute inflammation with necrosis (Figure 2). The inflammatory cell infiltrate included predominantly neutrophils, lymphocytes and occasional eosinophils. There was also some fibrinoid necrosis of blood vessels with extravasation of red blood cells. Fungal stains showed a negative reaction.

The patient was referred to a Rheumatologist and was started on prednisolone and cyclophosphamide. A few days after initiation of medical treatment, the patient felt a lot better and was discharged home for follow up.

## Discussion

The incidence of WG is 1 in 30,000. The Male:Female ratio is 1:1. It is an autoimmune disease affecting small/medium vessels and kidneys [2,3]. It is characterised by granulomas in the nose, sinuses, lungs, ear, eye and cranial/peripheral nerves. It was first described by Heinz Klinger, a German medical student in 1931. Later, Friedrich Wegener a German pathologist described 3 more cases and discovered it to be a vasculitis. WG is triggered by bacterial (*Staphylococcus aureus*) or viral (Parvo virus) infection. It is not hereditary. Therefore it is very unusual for WG to affect more than one member of the same family. It is an immune complex mediated or cell mediated segmental vasculitis [4]. Therefore negative biopsy does not exclude WG. It is characterised by the presence of granulomas which are localised microscopic collections of macrophages. In the lung, the granulomas may coalesce into masses which may cavitate [5]. Appendix 1 shows the criteria for diagnosing WG. Only a few cases of WG involving major arteries have been reported in the world literature [6] (Table 1). The affected arteries show fibrinoid necrosis [7]. There is no deposition of immunoglobulins within the kidney or vessel walls. When neutrophils are exposed to cytokines such as tumour necrosis factor, serine proteinase and myeloperoxidase are expressed on their surface. Anti neutrophilic antibodies against these cytokines damage the neutophils and release oxygen free radicals and intracellular enzymes which mediate the vasculitis.

**Table 1:** Studies showing involvement of major arteries in WG.

Ref	Patient details	Affected artery	Treatment	Outcome
[1]	63 year old male an inflammatory aortic aneurysm and polyneuropathy.	Aorta	Methyl prednisolone and trimethoprim-sulfamethoxazole	Good

[10]	34-year old Japanese man Pneumonia, paranasal sinusitis and clipping of a cerebral aneurysm	Anterior choroidal artery.	Prednisolone + cyclophosphamide	Good

[11]	67-year old man presented with abdominal pain and shock	Superior pancreatico-duodenal artery	Open repair of ruptured artery	Died from multi-organ failure

[4]	50-year old woman presented with abdominal pain and shock	Entire aorta(first intercostal artery to iliac bifurcation)	Was on steroids and cyclosphosphamide but could not prevent aortic dissection	Died from aortic dissection

[2]	58 year-old woman, pain in the upper limb	Subclavian aneurysm	Insertion of stent-graft and steroids	Good

[8]	Previous AAA patient	Aorta and subclavian	Details not known	

[7]	56-year-old Japanese man presented with shock	Ruptured left gastric	Aneurysm was diagnosed post mortem	Died of hemorrhagic shock

[3]	A hospitalized developed sudden hypovolemic shock	Ruptured hepatic artery aneurysm	Aneurysm was diagnosed post mortem	Died of hemorrhagic shock

[9]	Presented with respiratory and renal problems	Renal artery aneurysm	Aneurysm was diagnosed post mortem	Died

[5]	24 year old with massive perinephric haematoma	Bilateral renal artery aneurysm	Steroid and angioembolisation	Successfully recovered from the episode

[6]	29 year old with know WG presented with abdominal pain and vomiting	Renal and hepatic artery aneurysms	Steroid & angioembolisation	Successfully recovered

WG may affect the respiratory [8] and renal tracts [9]. It can also affect the eyes, skin, and peripheral nerves. Non-specific systemic symptoms are common. WG causes upper respiratory tract disease in > 90% of cases and causes sinusitis, nasal crusting, bleeding, obstruction and collapse of the nasal bridge. It can also cause otitis media and tracheal stenosis. When the lungs are affected, it may present with cough, haemoptysis and dyspnoea. Renal involvement may manifest as haematuria and proteinuria and can lead to renal failure. Ophthalmological manifestation includes sub conjunctival haemorrhages, scleritis, uveitis, keratitis, proptosis, or ocular muscle paralysis due to retro-orbital inflammation.

The disease doesn't affect the lymph nodes. Serology will be positive for anti-proteinase 3(Antineutrophil cytoplasmic antibody) which is highly specific for W G. The differential diagnosis for WG includes polyarteritis nodosa, Churg-Strauss, Henoch-Schonlein purpura, temporal arteritis and Takayasu syndrome (Table 2).

**Table 2:** Differential diagnosis for WG

Condition	Feature	Vascular involvement	Diagnosis	Comments
Churg-Strauss syndrome	Usually associated with asthma	Necrotising vasculitis affecting small to medium sized vessels	Eosinophilia and p-ANCA will be elevated	In WG c- ANCA will be high

Microscopic polyarteritis	Necrotising glomerulonephritis common	Mainly small vessel vasculitis	Negative for PR 3	Antiglomerular antibody will ne negative

Temporal arteritis	>50 years Head ache, jaw claudication	Granulomatous arteritis aorta and its major branches, especially extra cranial branches of carotid artery	Often affects temporal artery	Temporal artery biopsy is negative in 50%

Takayasu's arteritis	Absent upper limb pulses, systemic features such as fever, weight loss and joint pains	Granulomatous inflammation of aorta and its major branches Periaortitis, aortic dissection aneurysm and thrombosis of subclavian left gastric, hepatic and renal artery aneurysms	Diagnosis based upon American college of rheumatology criteria	Mainly affects Asian women

Ankylosing Spondylytis	Chronic back pain in young	5% can get aortitis Patchy destruction of both muscle and elastic tissue of media Fibrosis of intima	Radiologically there will sacroilitis	Seronegative arthropathy

The main treatment is immunosuppression and steroid therapy. A combination of cyclophosphamide and prednisone is effective [10] in > 90% with severe disease. Methotrexate and prednisone are useful when the disease spares the kidneys. Prophylactic antibiotics such as Septrin (Trimethoprim and Sulfamethoxazole) may prevent respiratory infections causing flare ups of WG. Untreated WG is fatal in 5 years [11]. Prednisone may slow progression of the disease but by itself is insufficient to arrest the disease.

## Conclusion

Whenever a young patient presents with an isolated aneurysm, dissection or aortitis screening for connective tissue disease should be performed. More than one vessel involvement in a young patient should alert the clinician to exclude a systemic disease. Early steroid therapy and immunosuppression may alter the course of the disease and hence its prognosis.

## Abbreviations

IJV: internal jugular vein; WG: Wegener's granulomatosis.

## Consent

Written informed consent was obtained from the patient for publication of this case report and accompanying images. A copy of the written consent is available for review by the Editor-in-Chief of this journal.

## Competing interests

The authors declare that they have no competing interests.

## Authors' contributions

RD wrote the manuscript, RA and KP are responsible for the pathology section and slides, KB supervised RD in preparation of the manuscript, All authors read and approved the final manuscript.

## Appendix 1 - Criteria for diagnosing WG

1) Painful or painless oral ulcers or purulent or bloody nasal discharge

2) Chest radiograph showing the presence of nodules, fixed infiltrates, or cavities

3) Haematuria (>5 red blood cells per high power field) or red cell casts in urine sediment

4) Histological changes showing granulomatous inflammation within the wall of an artery or in the perivascular or extra vascular area (artery or arteriole)
